# Immunohistochemical assessment of a unique basal pattern of p53 expression in ulcerative-colitis-associated neoplasia using computer-assisted cytometry

**DOI:** 10.1186/1746-1596-9-99

**Published:** 2014-05-29

**Authors:** Shunsuke Kobayashi, Takahiro Fujimori, Hiroyuki Mitomi, Shigeki Tomita, Kazuhito Ichikawa, Johji Imura, Shigehiko Fujii, Michihiro Itabashi, Shingo Kameoka, Yoshinori Igarashi

**Affiliations:** 1 Department of Surgical and Molecular Pathology, Dokkyo Medical University School of Medicine, 880 Kitakobayashi, Mibu, Shimotsuga, Tochigi 321-0293, Japan; 2 Division of Gastroenterology and Hepatology, Department of Internal Medicine, Toho University Omori Medical Center, 6 - 11-1, Omorinishi, Ota - ku, Tokyo 143-8541, Japan; 3 Department of Diagnostic Pathology, Graduate School of Medicine and Pharmaceutical Sciences, University of Toyama, 2630 Sugitani, Toyama 930-0194, Japan; 4 Center for Gastrointestinal Endoscopy, Kyoto-Katsura Hospital, 17 Yamada-Hirao, Nishikyo, Kyoto 615-8256, Japan; 5 Department of Surgery 2, Tokyo Women’s Medical University, 8-1 Kawada-cho, Shinjuku, Tokyo 162-8666, Japan

**Keywords:** Ulcerative colitis, p53, Immunohistochemistry, Computer-assisted cytometry, Dysplasia

## Abstract

**Background:**

The basal pattern of p53 expression, defined as its immunoreactivity confined to the basal half of the glands, is associated with early neoplastic lesions in ulcerative colitis (UC). However, their clinical utility of this finding is limited by the use of “visual estimation” (approximate immunoreactivity on the basis of scanning the stained slide, without formal counting). This study was designed to analyze the basal pattern of p53 using computer-assisted cytometry and to identify the optimal cutoff value for discriminating between UC-associated early-stage neoplasia and regenerative atypia.

**Methods:**

The specimens were obtained from eight UC patients undergoing colectomy and were classified according to the criteria by the Research Committee of Inflammatory Bowel Disease of the Ministry of Health and Welfare in Japan. Patients with classes UC-IIa (indefinite for dysplasia, probably regenerative), UC-IIb (indefinite for dysplasia, probably dysplastic), and UC-III (definitive dysplasia) were enrolled in the study. Based on the percentage of immunoreactive cells in the basal half of the crypt with visual estimation, basal positivity of p53 was classified into three categories: grade 1 (1 - 9%), grade 2 (10 - 19%), and grade 3 (≥20%). Next, crypts classified as grade 3 by visual estimation were analyzed by computer-assisted image analysis.

**Results:**

Using visual estimation, grade-3 p53 basal positivity was observed in 46.0% of UC-IIa crypts (128 of 278), 61.9% of UC-IIb crypts (39 of 63), and 94.2% of UC-III crypts (81 of 86). Using image analysis, the median p53 basal positivities were 30.3% in UC-IIa, 52.3% in UC-IIb, and 65.4% in UC-III (*P* ≤0.002). A receiver operating characteristics curve was generated to determine the method’s diagnostic utility in differentiating UC-IIa from UC-III. In this cohort, the sensitivity was 0.78; the specificity was 0.98; the negative predictive value was 87.4%; the positive predictive value was 95.5%, and the accuracy was 90.2% with a cutoff value for p53 basal positivity of 46.1%.

**Conclusions:**

Our findings indicate that assessing p53 basal positivity by image analysis with an optimal threshold represents an alternative to visual estimation for the accurate diagnosis of UC-associated early-stage neoplasia.

**Virtual Slides:**

The virtual slide(s) for this article can be found here: http://www.diagnosticpathology.diagnomx.eu/vs/3588120501252608

## Background

There is an increasing incidence of ulcerative colitis (UC)-associated neoplasia among patients with long-standing and extensive disease [1,2]. However, UC-associated early-stage neoplasia is not easily detected by endoscopy, because those lesions are sometimes missed among the inflamed epithelium [3]. In addition, the histological diagnosis of early-stage neoplasia, which often has unremarkable cytological alterations [4], is difficult due to the recurrent and persistent inflammatory changes associated with UC. Other difficulties in early detection result from specific histological features of UC-associated early-stage neoplasia, which can involve only one part of the crypt or a few crypts in the inflamed epithelium [4]. Consequently, it is difficult to distinguish UC-associated early-stage neoplasia from regenerative atypia in biopsy specimens stained with hematoxylin and eosin (H&E) [5]. Moreover, there is a concerning degree of inter- and intra-observer variation in the diagnosis of UC-associated early-stage neoplasia and regenerative atypia among gastrointestinal pathologists [5].

Mutations in the *p53* gene represent an early event in the development of UC-associated neoplasia [6-8]. Immunohistochemical analysis of the p53 protein is a useful method for detecting UC-associated early-stage neoplasia, since there is a concordance between *p53* gene mutation and its expression [7-9]. Immunohistochemstry for p53 was positive in 11 - 75% of low-grade dysplasia and 45 - 83% of high-grade dysplasia but was negative in regenerative epithelium in UC [9-12]. Accordingly, Wong *et al*. reported intense p53 staining in 14% of low-grade dysplasia and 57% of high-grade dysplasia but not in regenerative epithelium with atypia in UC [13]. They reported that restriction of p53 expression to the basal two-thirds of the crypt excluded a diagnosis of UC-associated high-grade dysplasia. Noffsinger *et al.* reported that p53 basal expression was associated with *p53* mutation and represented an early neoplastic change in a subgroup of UC patients [7]. Moreover, UC-associated early-stage neoplasia had a significantly higher proportion and a different pattern of p53 immunoreactivity than sporadic adenoma [11,14]. The immunoreactivity in sporadic adenomas had a scattered or sporadic pattern of p53 expression [11], while UC-associated early-stage neoplasia exhibited a “bottom-up” pattern [7,13]. However, the utility of these reports is limited by the “visual estimation” of basal pattern of p53 immunoreactivity, where the approximate immunoreactivity was determined by scanning the stained slide without formal counting [15]. Recently, the emergence of digital pathology and software analysis of data has led to the development of a reliable and reproducible digital analytical technique for Ki-67 immunoreactivity [15,16].

The aim of this study was to analyze the basal pattern of p53 immunoreactivity using computer-assisted cytometry and to identify the optimal cutoff value for basal positivity of p53 in order to discriminate between UC-associated early-stage neoplasia and regenerative atypia.

## Methods

### Patients and materials

Tissue samples were obtained from eight UC patients (five males and three females; mean/median age, 46.0/47.5 years, range, 21–67 years; mean/median duration of disease, 21.3/15.0 years, range 6–43 years) who underwent total colectomy for UC-associated neoplasia at Tokyo Women’s Medical University and Dokkyo Medical University School of Medicine between January 2010 and December 2011.

Colectomy specimens were fixed in 10% formalin and cut to a width of 5 mm and then embedded in paraffin, sectioned, and stained with H&E. The specimens were histologically evaluated by three experienced gastrointestinal pathologists (S.K., T.F., H.M.) and were classified according to the classification proposed by the Research Committee of Inflammatory Bowel Disease of the Ministry of Health and Welfare in Japan [17]. Inter-observer variations were resolved by reevaluation and discussion to reach consensus. Patients classified as having UC-IIa (indefinite for dysplasia, probably regenerative), UC-IIb (indefinite for dysplasia, probably dysplastic), and UC-III (low or high-grade dysplasia) were enrolled in this study.

The Ethics Committee of Dokkyo Medical University School of Medicine approved all protocols, and informed consent for tissue procurement was obtained from all patients. Samples used in this study were surgically resected for treatment, not for research purposes. Participation in the present study did not increase medical disadvantage or risk to patients.

### Immunohistochemistry for p53

Briefly, 4-μm-thick serial sections were placed on silane-coated slides, deparaffinized, rehydrated, and pretreated with 0.3% hydrogen peroxidase in methanol at room temperature to quench endogenous peroxidase activity. The sections were then placed in 0.01 M citrate buffer (pH 6.0) and treated by microwave heating (MI-77, Azumaya, Tokyo, Japan; 400 W, 95°C) to facilitate antigen retrieval. The sections were incubated with 1% bovine serum albumin in phosphate-buffered saline and then with a monoclonal anti-human p53 antibody (DO-7; 1:50 dilution; DAKO, Glostrub, Denmark). Immunohistochemical staining was performed with EnVisionTM + Kits (DAKO). Finally, the sections were incubated with 3,3′-diaminobenzidine and counterstained lightly with Carazzi’s hematoxylin.

### Visual estimation of p53 expression

Neoplastic lesions in each p53-immunostained slide were confirmed using the corresponding H&E-stained specimens. We evaluated the unique basal pattern of p53 expression, which was characterized by p53 immunoreactivity in the basal half of the crypts, as described by Noffisinger *et al*. [7]. In addition, the unique basal pattern of p53 expression was classified into three categories according to the percentage of immunoreactive cells in the basal half of the crypt as follows: grade 1 (1 - 9%); grade 2 (10 - 19%), and grade 3 (≥20%). Immunohistochemical images were imported into a PowerPoint presentation and evaluated by two independent researchers (T.F. and S.K.), who estimated the grade of p53 immunoreactivity based on their inspection of the images without using computer-assisted image analysis (‘visual estimation’). Inter-observer disagreements were resolved by reevaluation and discussion to a reach consensus.

### Analysis of p53 expression using computer-assisted cytometry

Crypts classified as grade 3 by visual estimation were analyzed using the computer-assisted cytometry with WinROOF image-processing software (Mitani Corp., Tokyo, Japan). The glands subjected to image analysis were continuously visible from the bottom of the glands to the surface epithelium. The manual counting function of the image analysis was utilized, and p53-positive cells in the basal half of the crypts were counted under high-power magnification (at 400X) using a touch pen by introducing a liquid crystal touch panel. The values were expressed as the percentage of p53-positive cells relative to the total number of crypt cells in the basal half of the glands.

### Statistical analysis

Continuous data were analyzed with the Mann–Whitney *U*-test using Stat Flex Version 6.0 (Artec Co. Ltd., Osaka, Japan). A *P*-value < 0.05 was considered statistically significant. A receiver operating characteristics (ROC) curve was generated with *R* Version 3.0.1 statistical software.

## Results

A total of 1,039 crypts (a total of 103 lesions) consisting of 626 UC-IIa crypts (total 40 lesions; Case #1, 13 lesions; Case #3, 1 lesion; Case #4, 1 lesion; Case #7, 12 lesions; Case #8, 13 lesions), 256 UC-IIb crypts (40 lesions; Case #1, 10 lesions; Case #2, 6 lesions; Case #3, 10 lesions; Case #4, 6 lesions; Case #5, 4 lesions; Case #6, 4 lesions), and 157 UC-III crypts (23 lesions; Case #1, 7 lesions; Case #2, 9 lesions; Case #3, 3 lesions; Case #5: 3 lesions; Case #6, 1 lesion) were examined by visual estimation.

In first visual estimation, 278 out of 626 crypts (44.4%; captured for 4 cases) in UC-IIa, 63 of 256 crypts (24.6%; captured for 5 cases) in UC-IIb, and 86 of 157 crypts (54.8%; captured for 4 cases) in UC-III revealed basal positivity for p53. Grade-3 p53 basal positivity was observed in 128 of 278 crypts (46.0%) in UC-IIa, 39 of 63 crypts (61.9%) in UC-IIb, and 81 of 86 crypts (94.2%) in UC-III (Table 1).

**Table 1:** Grade of p53 basal positivity in the crypts in UC-IIa, IIb, and III lesions

**Grade of p53 basal positivity**	**UC-IIa (%)**	**UC-IIb (%)**	**UC-III (%)**
	**(n = 278)**	**(n = 63)**	**(n = 86)**
Grade 1	74 (26.6)	16 (25.4)	2 (2.3)
Grade 2	76 (27.3)	8 (12.7)	3 (3.5)
Grade 3	128 (46.1)	39 (61.9)	81 (94.2)

**Figure 1:**
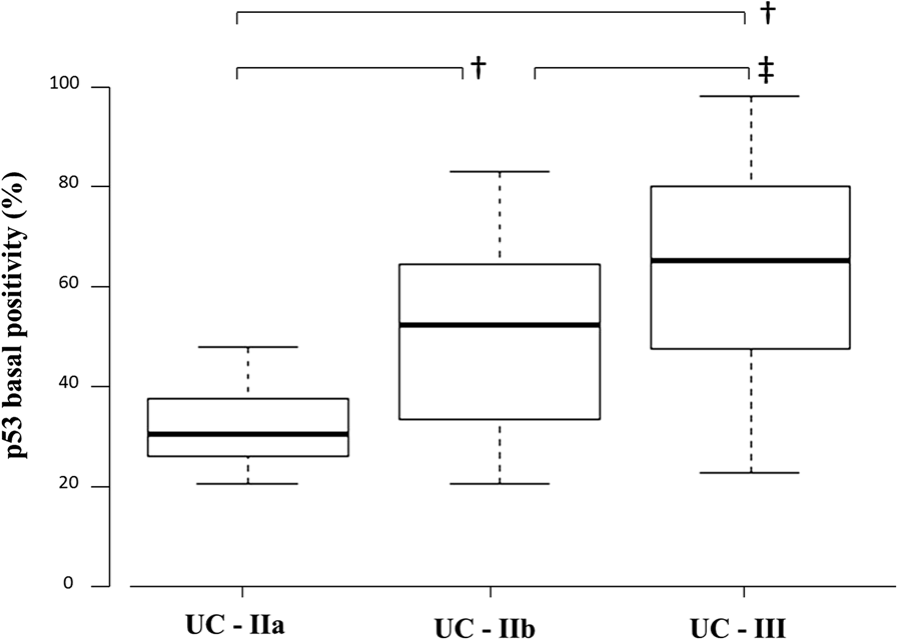
**p53 basal positivity in grade 3 UC-IIa, IIb, and III.** Median values of the p53 basal positivities were 30.3% in UC-IIa, 52.3% in UC-IIb, and 65.4% in UC-III with statistically significant differences among the all categories. Middle lines, median; boxes, 25th to 75th percentiles; upper limit, 95th percentile; lower limit, 10th percentile; †, *P* < 0.001; ‡, *P =* 0.002.

**Figure 2:**
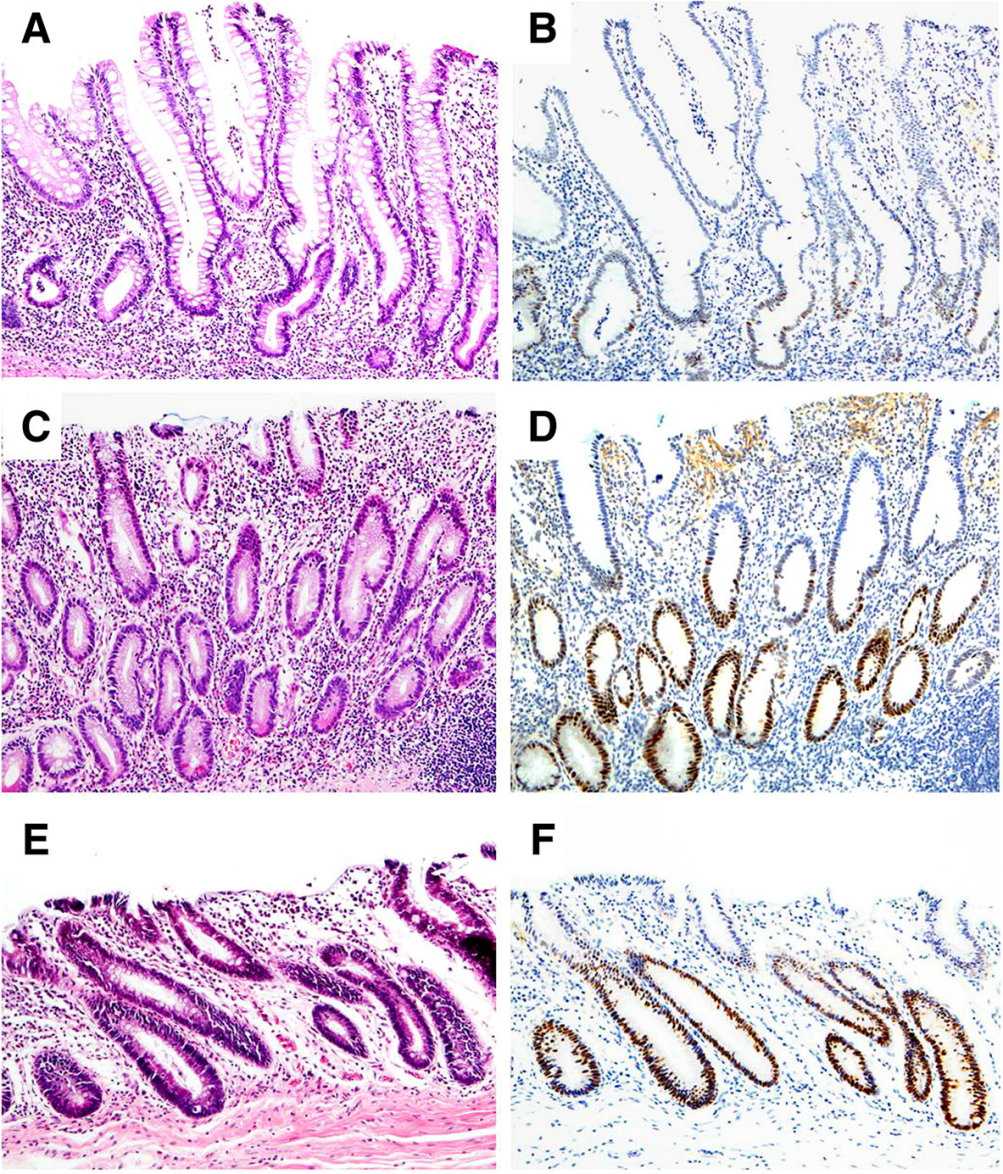
**Basal positivity of p53 in representative cases of UC-IIa, IIb, and III. (A)** UC-IIa showing mild glandular distortion with slight nuclear enlargement and hyperchromatism at the bottom of crypts. There is no nuclear stratification or mucin depletion. Moderate small round cell infiltration with crypt abscess is present in the lamina mucosae (H&E, original magnification × 10). **(B)** Weak intensity of p53 staining is restricted to the lower third of the crypts (serial sections of ‘**A**’; immunoperoxidase, original magnification × 10). **(C)** UC-IIb demonstrating hyperchromatic and enlarged nuclei with slight stratification and occasional goblet cell depletion in the lower half of the crypts. Mild to moderate inflammation is seen in the background mucosa (H&E, original magnification × 10). **(D)** p53 positivity is limited to the lower half of the crypts (serial section of ‘**C**’; immunoperoxidase, original magnification × 10). **(E)** UC-III shows marked hyperchromatic and stratified nuclei with some variation in size and shape from the bottom of the crypt to the surface epithelium. Marked goblet cell depletion with mild inflammation is present in the mucosa (H&E, original magnification × 10). **(F)** Strong p53 expression is identified in the lower two thirds of the crypts (serial section of ‘**E**’; immunoperoxidase, original magnification × 10).

For samples with grade-3 p53 basal positivity by qualitative estimate, computer-assisted cytometry was performed using the WinROOF image-processing software. In this analysis, median p53 basal positivities were 30.3% (25–75 percentile: 25.6 - 37.6%) in UC-IIa, 52.3% (33.3 - 64.7%) in UC-IIb, and 65.4% (47.2 - 80.6%) in UC-III. There were statistically significant differences between IIa and IIb (*P* < 0.001), IIa and III (*P* < 0.001), and IIb and III (*P* = 0.002) (Figure 1). Basal positivity of p53 in representative cases of UC-IIa, IIb, and III lesions are illustrated in Figure 2.

**Figure 3:**
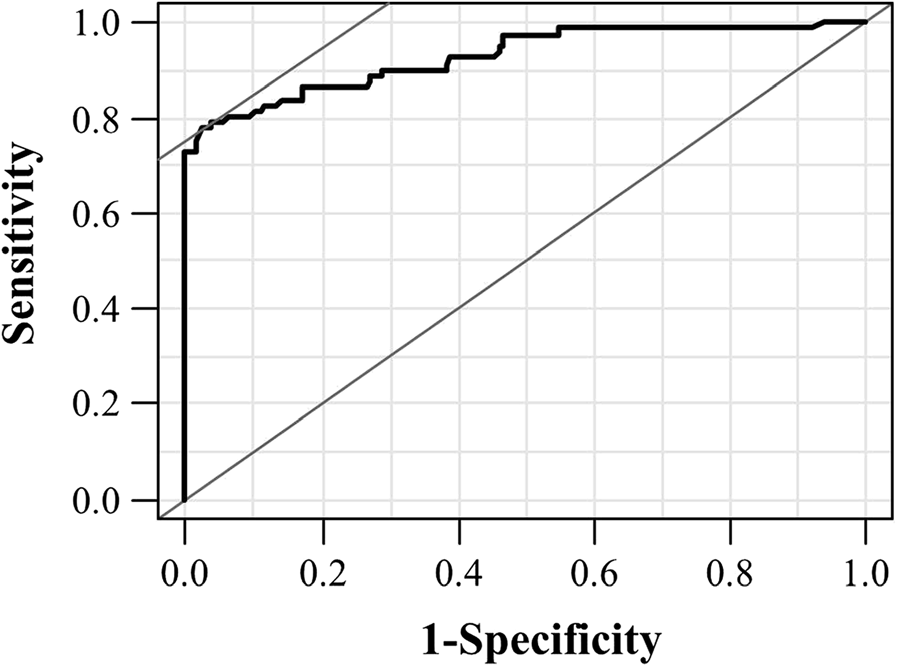
**ROC curve illustrating the ability to differentiate between UC IIa and UC-III.** ROC curve illustrating the high diagnostic ability with a cutoff value of 46.1% for p53 basal positivity in the differentiation of UC-III *vs.* UC-IIa, AUC = 0.928 (95% CI, 41.48 - 47.11; *P* < 0.001).

We generated the ROC curve in order to determine our ability to differentiate between UC-IIb and UC-III, but the sensitivity and negative predictive value and accuracy were low (area under the curve [AUC], 0.678; 95% confidence interval [CI], 55.82 - 63.18; *P* = 0.002; sensitivity, 0.42; specificity, 0.872; negative predictive value, 42.0%; positive predictive value, 87.2%; accuracy, 56.7%). We subsequently determined the ability to differentiate between UC-IIa and UC-III by the ROC curve (Figure 3). The AUC was 0.928 (95% CI, 41.48 - 47.11; *P* < 0.001); the sensitivity was 0.78; the specificity was 0.98: the negative predictive value was 87.4%; the positive predictive value was 95.5%, and the accuracy was 90.2% with a cutoff value of 46.1% for p53 basal positivity. Using this threshold, 2.3% of UC-IIa crypts (3 of 128) and 61.5% of UC-IIb crypts (24 of 39) corresponded to UC-III, whereas 22.2% of UC-III crypts (18 of 81) were excluded from being UC-III (Table 2).

**Table 2:** Frequency of UC-IIa, IIb, and III based on a new p53 basal positivity cutoff value

**p53 basal positivity**	**UC-IIa (%)**	**UC-IIb (%)%**	**UC-III (%)**
	**(n = 128)**	**(n = 39)**	**(n = 81)**
20.0 - 46.0	125 (97.7)	15 (38.5)	18 (22.2)
>46.1	3 (2.3)	24 (61.5)	63 (77.8)

## Discussion

In the present study, we investigated the basal pattern of p53 immunoreactivity in UC-III (definite dysplasia) and UC-II (indefinite for dysplasia) crypts using computer-assisted cytometry and determined the optimal cutoff value for basal positivity of p53 for discriminating between the two categories. By visual estimation, Grade-3 p53 basal positivity was observed in 46.0% of UC-IIa crypts (indefinite for dysplasia, probably regenerative), 61.9% of UC-IIb crypts (indefinite for dysplasia, probably dysplastic), and 94.2% of UC-III crypts. Our data are consistent with the previously published hypothesis that the basal pattern of p53 expression is not a specific sign of dysplasia (UC-III); rather, this pattern was observed in 5 - 64% of regenerative mucosa and 13 - 33% of samples indefinite for dysplasia [7,13].

There is significant discordance between visual estimation and digital image analysis for Ki-67 immunoreactivity [15]. Thus, we performed computer-assisted cytometrical analysis using the WinRooF image-processing software, which we developed in our previous study on Ki-67 immunoreactivity [16], for grade-3 p53 basal positivity by visual estimation. We could exclude non-neoplastic (dysplastic) cells that occasionally display nonspecific positivity for p53, such as lymphocytes or histiocytes, using a touch pen by introducing a touch panel. Using this analysis, we identified that p53 basal positivity was significantly higher in UC-III crypts (median value, 65.4%) than in UC-IIb crypts (52.3%) or UC-IIa crypts (30.3%). However, the ROC curve indicated a low sensitivity (0.42) and negative predictive value (42.0%) in differentiating between UC-III and UC-IIb. Based on this result, we considered the possibility that some of the UC-IIb crypts might be included in the UC-III category. Indeed, 80% of UC-IIb crypts harbored *p53* mutations [8].

Colectomy is often indicated in patients with a diagnosis of UC-III, whereas those with UC-IIa should undergo repeat surveillance colonoscopy within a short interval [2], but there is poor inter-observer agreement for the two categories [5]. In this line, Matalka *et al.* developed an automated intelligent system to quantitatively assess inflammatory bowel system, which proved to be reliable and minimizes inter- and intraobserver variability [18]. In our ROC analysis for UC-III and UC-IIa showing p53 basal positivity, the sensitivity (0.78) and negative predictive value (87.4%) were improved when the cutoff value was 46.1%. At this threshold, only 2.3% of UC-IIa, but 61.5% of UC-IIb crypts were classified as UC-III.

A major goal in screening for definitive dysplasia is minimizing the false negative rate; therefore, we identified a threshold of p53 basal positivity (*i.e.*, > 40%) at which 17.2% of UC-IIa crypts were interpreted as UC-III, and only 13.6% of UC-III crypts were missed from definitive dysplasia. This indicates moderate-to-high sensitivity (86.4%) and specificity (82.8%). These values are consistent with the finding that 20% of UC-IIa crypts and 92% of UC-III crypts harbored *p53* mutations [8]. When p53 basal positivity is above this threshold, even if the biopsy diagnosis is morphologically indefinite for early-stage neoplasia in H&E stained biopsy specimens, we recommend genetic analysis for *p53* or appropriate treatment, such as endoscopic or surgical resection.

## Conclusions

Our present findings suggest that using computer-assisted cytometrical analysis of p53 basal positivity with an optimal threshold (standardization) represents an alternative to visual estimation as an accurate tool for detecting UC-associated early-stage neoplasia. The choice of an appropriate therapeutic strategy may be informed by using p53 basal positivity in biopsy samples in UC patients.

## Competing interests

The authors declare that they have no competing interests.

## Authors’ contributions

Study concept and design: TF and SK; immunohistochemical analysis: SK, TF and HM; acquisition of data: SF, MI, and SK; analysis and interpretation of data: ST and KI; drafting of the manuscript: SK, TF, and HM; critical revision of the manuscript for intellectual content: JI and ST; study supervision: YI and TF. All authors read and approved the final manuscript.
